# On Sandy, Boron-Poor Soils, Liming Induced Severe Boron Deficiency and Drastically Reduced the Dry Matter Yield of Young Olive Trees

**DOI:** 10.3390/plants12244161

**Published:** 2023-12-14

**Authors:** Margarida Arrobas, Soraia Raimundo, Nuno Conceição, José Moutinho-Pereira, Carlos Manuel Correia, Manuel Ângelo Rodrigues

**Affiliations:** 1 Centro de Investigação de Montanha (CIMO), Instituto Politécnico de Bragança, Campus de Santa Apolónia, 5300-253 Bragança, Portugal; marrobas@ipb.pt (M.A.); sraimundo@ipb.pt (S.R.); nuno.conceicao@ipb.pt (N.C.); 2 Laboratório para a Sustentabilidade e Tecnologia em Regiões de Montanha, Instituto Politécnico de Bragança, Campus de Santa Apolónia, 5300-253 Bragança, Portugal; 3 Centre for the Research and Technology of Agro-Environmental and Biological Sciences (CITAB), University of Trás-os-Montes and Alto Douro, 5001-801 Vila Real, Portugal; moutinho@utad.pt (J.M.-P.); ccorreia@utad.pt (C.M.C.)

**Keywords:** soil acidity, *Olea europaea*, olive cultivars, schist soil, granite soil, phosphorus fertilization

## Abstract

In the northeast of Portugal, like in many parts of the world, most soils are acidic, which may hamper crop productivity. This study presents the findings of a factorial experiment on olive (*Olea europaea* L.) involving three factors: (i) soil type [schist (Sch) and granite (Gra)]; (ii) cultivars [Cobrançosa (Cob) and Arbequina (Arb)]; and (iii) fertilizer treatments [liming (CaCO_3_) plus magnesium (Mg) (LMg), phosphorus (P) application (+P), boron (B) application (+B), all fertilizing materials combined (Con+), and an untreated control (Con-)]. Dry matter yield (DMY) did not show significant differences between cultivars, but plants grown in schist soil exhibited significantly higher biomass compared to those in granite soil. Among the treatments, +B and Con+ resulted in the highest DMY (50.8 and 47.2 g pot^−1^, respectively), followed by +P (34.3 g pot^−1^) and Con- (28.6 g pot^−1^). Treatment LMg yielded significantly lower values (15.6 g pot^−1^) than Con-. LMg raised the pH above 7 (7.36), leading to a severe B deficiency. Although Con+ also raised the pH above 7 (7.48), it ranked among the most productive treatments for providing B. Therefore, when applying lime to B-poor sandy soils, moderate rates are advised to avoid inducing a B deficiency. Additionally, it seems prudent to apply B after lime application.

## 1. Introduction

Soil acidity is an ecological factor that affects the growth and yield of many crops. It is estimated that approximately 30% of the world’s total ice-free land and 50% of potentially arable land is affected by topsoil acidity [1,2,3]. The greatest occurrence of acidity is reached in humid regions, where rainfall exceeds evapotranspiration. Thus, leading to the leaching of base cations, such as calcium (Ca^2+^), magnesium (Mg^2+^) and potassium (K^+^), which are replaced by hydronium (H_3_O^+^) and aluminum (Al^3+^) ions [3,4]. Long-term cultivation may also increase soil acidity, since plants usually take up more cations [e.g., Ca^2+^, Mg^2+^, K^+^, ammonium (NH_4_^+^)] than anions [e.g., nitrate (NO_3_^−^), sulfate (SO_4_^2^)], thereby exuding hydrogen ions (H^+^) into the soil solution to maintain charge balance [5]. Other anthropogenic processes can lower soil pH, such as the use of ammonium fertilizers and some manures and composts. Soil pH decreases due to the oxidation of ammonium N and organic and inorganic acids formed during the decomposition of organic substrates [4,6].

The toxicity of Al and manganese (Mn) are the most important factors that restrict plant establishment in acidic soils [2,5]. Toxicity caused by H^+^ only occurs in very acidic soils, generally below pH 3 [4,7]. Acid soils are also often associated with Ca, Mg and P deficiencies [1,5,8]. In fact, soil pH is one of the primary regulators of nutrient cycling in the soil, influencing the availability of many other nutrients such as molybdenum (Mo), iron (Fe), zinc (Zn) and copper [5,8].

Liming is used to reduce or neutralize soil acidity. The application of lime increases soil pH and this reduces the concentration of Al^3+^ and Mn^3+^ in the soil [4,5]. Furthermore, liming increases exchangeable Ca^2+^ and also Mg^2+^ if dolomitic limestone is used [9,10]. Changing the bioavailability of these and many other nutrients (e.g., P, Fe, Zn, Cu) by liming creates more favorable conditions for plant growth [10].

In the Northeast of Portugal, the main lithological formations are schist and granite [11]. These lithological substrates have low levels of alkaline cations, mainly Ca^2+^ and Mg^2+^ [12,13]. The region benefits from a Mediterranean climate, with two well-defined seasons, the cold and wet winter, and the hot and dry summer [14]. Precipitation is concentrated in winter when evapotranspiration is minimal, generating a surplus of rain that percolates into the soil washing out base cations. On the other hand, the soil’s resistance to pH change depends on its buffering capacity and this, in turn, on the content of organic matter and 2:1 clay minerals [5]. The region consists of a landscape of steep gradients. The soils are shallow and heavily eroded, with very low levels of organic matter and clay, aspects that make them very prone to acidity [12,13]. The soils in this region also have low or very low phosphorus (P) levels [12]. Although the primary limitations in acid soils are toxic levels of Al and Mn, suboptimal levels of P are usually also of great concern and a major objective of liming [1,6]. In this region in particular, acidic soils also provide low levels of boron (B) to crops [15].

Although it is a micronutrient, B has a role in the fertilization of dicotyledonous species in the region equivalent to the application of N [15]. Several studies have shown that soils have low levels of B, with plants typically experiencing a high response to its application [15,16,17]. The olive tree is one of the most economically important crops in the northeast of Portugal. In general, the species is considered to adapt to a wide range of soil pH [18,19]. In the northeast of Portugal, one of the most common cultivars is Cobrançosa, which has been grown quite successfully in highly acidic soils [13]. On the other hand, many other cultivars, such as Arbequina, are grown in Southern Europe and North Africa [19,20], in arid and semi-arid regions where neutral to alkaline soils normally predominate [3,5]. Perhaps the species’ adaptability to pH results from the high diversity of cultivars grown in different olive growing regions [10].

Therefore, the objective of this study is to evaluate the olive tree’s response to liming and/or application of P and B. To create experimental contrast, two cultivars were used: Cobrançosa, which is known for being well adapted to acidic and low-fertility soils [14]; and Arbequina, generally grown in neutral to alkaline soils with high-cropping intensification [21]. Two very low pH (<5) soils were also used, schist and granite. The study also included five fertilizer treatments, (i) application of calcium carbonate (CaCO_3_), to raise the pH and provide Ca, supplemented with application of Mg; (ii) application of P; (iii) application of B; (iv) a positive control that received all the fertilizers mentioned above; and (v) a negative control that did not receive any fertilizer. The main hypothesis put forward is that increasing pH can improve plant performance. Secondary hypotheses are that the application of P and/or B alone may increase plant growth, given the generally recognized relevance of these nutrients in the fertilization of crops.

## 2. Results

### 2.1. Dry Matter Yield

**Figure 1:**
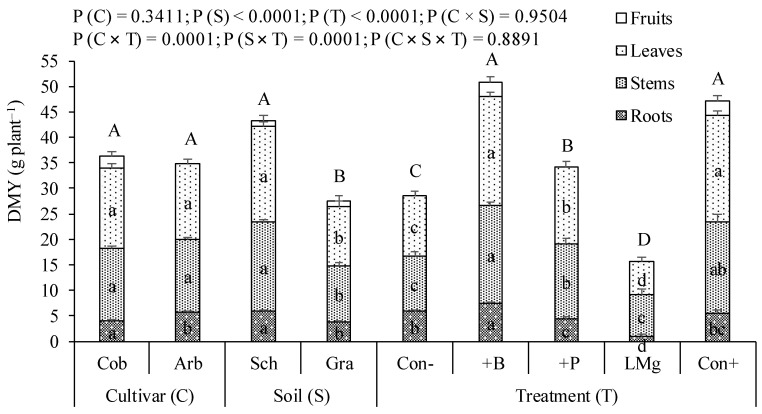
Dry matter yield (DMY) of olive plants of the cultivars Cobrançosa (Cob) and Arbequina (Arb), grown in schist (Sch) and granite (Gra) derived soils under five fertilizer treatments [untreated control (Con-), boron (+B) and phosphorus (+P) application, calcium carbonate plus magnesium application (LMg), and positive control receiving all these fertilizers (Con+)]. Within cultivar, soil or treatment, means followed by the same letter are not significantly different by the Tukey HSD test (α = 0.05). Lowercase letters compare the different plant parts (leaves, stems or roots) and capital letters total DM yield. Error bars are the standard error.

Total dry matter yield (DMY) did not vary significantly between the two cultivars, Cobrançosa and Arbequina, used in this study (Figure 1). However, Cobrançosa bore the first fruits (2.66 g plant^−1^) during the experimental period, while Arbequina did not produce any fruits. The schist soil provided a significantly higher total DMY compared to the granite soil, as a result of the increased values of the three parts of the plant, roots, stems and leaves. The +B and Con+ treatments presented DMY significantly higher than all other treatments. The P treatment, in turn, provided a significantly higher DMY than Con-, while the liming plus Mg (LMg) treatment provided a lower DMY than the latter. The roots and also the leaves seemed to be the parts of the plant most negatively affected by LMg treatment. Fruits appeared only in treatments that received B (+B and Cont+), which also coincided with treatments that produced more total biomass.

### 2.2. Nutrient Concentration in Plant Tissues

Leaf N concentration was significantly higher in Arbequina (17.0 g kg^−1^) compared to Cobrançosa (15.8 g kg^−1^) (Table 1). However, the result was not consistent between plant tissues, with the stems (Table 2) and the roots (Table 3) of Cobrançosa showing significantly higher levels of N. Plants that grew in the schist-derived soil showed significantly higher N concentrations in all the tissues compared to those grown in the granite soil. Leaf N concentration also varied significantly between fertilizer treatments. Con-, the only treatment that did not receive N as a fertilizer, showed the lower values. The result was consistent with those of N concentration in the stems and roots. The treatments producing more biomass, +B and Con+, also showed a consistent trend towards lower concentration of N in all plant parts.

The concentrations of P in leaves (Table 1) and stems (Table 2) were significantly higher in Arbequina compared to Cobrançosa. However, the opposite occurred in the roots, where the values were significantly higher in Cobrançosa than in Arbequina (Table 3). The concentrations of P were significantly higher in all plant tissues in plants grown in soil derived from granite. Fertilizer treatments did not significantly influence the levels of P in plant tissues.

K concentration in leaves (Table 1), stems (Table 2) and roots (Table 3) did not vary significantly between cultivars or soil types. Furthermore, no significant differences were found between fertilizer treatments in K concentration in leaves and stems. However, significant differences in root K concentrations were found between treatments, with a tendency to lower values in treatments that produce more biomass and also fruits on the Cobrançosa cultivar.

Leaf Ca concentration did not vary significantly between cultivars (Table 1), but the levels of Ca in stems (Table 2) and roots (Table 3) were significantly higher in Cobrançosa. The soils had a reduced influence on the Ca concentration in the tissues; only in the stems did significant differences appear, with higher values occurring in the schist soil. Fertilizer treatments significantly influenced the Ca concentration in all plant tissues, with a tendency for treatments that received CaCO_3_ to show higher Ca levels.

Arbequina showed significantly higher and lower Mg levels in the shoots (Table 1 and Table 2) and in the roots (Table 3), respectively, compared to Cobrançosa. The soil type also significantly influenced the concentration of Mg in the shoots, with schist soil showing the highest values. Fertilizer treatments had little influence on the Mg levels in the tissues. Only in the roots were significant differences between treatments observed, with the highest values appearing in treatments that received Mg (LMg and Con+) or that did not receive K (Con-).

Arbequina tended to have higher B levels in the tissues than Cobrançosa, with significant differences in the values observed in the stems (Table 1, Table 2 and Table 3). Plants grown in granite-derived soil also had higher B concentrations than those grown in schist, with significant differences in leaves and stems. B in tissues also varied significantly with fertilizer treatments. The treatments that received B (+B and Con+) presented the highest values. The lowest values tended to appear in the +P treatment.

Arbequina showed higher Fe concentrations in all plant tissues than Cobrançosa (Table 1, Table 2 and Table 3). The soil, in contrast, had a less evident effect on the Fe levels in the tissues, only in the stems were the Fe concentrations in the granite soil higher than those in the schist soil. The fertilized treatments receiving CaCO_3_ showed lower Fe levels in the leaves and a similar trend for the other tissues.

Leaf Mn concentrations varied significantly with cultivar, soil type and fertilizer treatment (Table 1). Arbequina plants, grown on granite soil and receiving CaCO_3_ showed lower Mn levels in the leaves. The other plant tissues showed a pattern not greatly dissimilar to the leaves (Table 2 and Table 3).

The effect of cultivar on Zn concentration in tissues depended on the tissue itself (Table 1, Table 2 and Table 3). Zn appeared in higher and lower concentrations in the shoot and root, respectively, in the Cobrançosa cultivar, compared to Arbequina. The effect of soil type also seemed inconsistent. Plants grown in schist soil had higher Zn levels in leaves, but lower levels in stems and roots. The effect of treatments on Zn levels in tissues also did not seem very enlightening. However, a general assessment based on the three types of tissues analyzed seems to indicate that treatments that received CaCO_3_ present the lowest values.

Tissue Cu concentrations were significantly higher in Arbequina than in Cobrançosa (Table 1, Table 2 and Table 3). Plants grown in granitic soil also showed a consistent trend towards higher Cu values compared to those found in plants grown in schist soil. Fertilizer treatments receiving CaCO_3_ showed a general trend towards lower Cu levels in plant tissues.

The concentration of Al was also determined in the roots (Table 3). There were no significant differences between cultivars or soil type. The effect of the treatments on the concentration of Al in the roots was significant, with the values being lower in the treatments that received CaCO_3_ (LMg and Con+).

### 2.3. Soil Properties

Soil organic C varied significantly with cultivar and soil type (Table 4). The pots grown with Cobrançosa showed higher levels of soil organic C. Schist soil also showed significantly higher levels of organic C. Fertilizer treatments did not influence significantly soil organic C. Soil pH at the end of the experimental period did not vary with cultivar or soil type, but varied significantly with fertilizer treatments. The pots receiving CaCO_3_ showed higher pH values. Extractable P was significantly higher in the pots grown with Cobrançosa and in granitic soil. The treatments receiving P (+P and Con+) and those receiving CaCO_3_ (LMg and Con+) showed higher levels of extractable P. The pots where Arbequina was grown and schist soil showed K values in the soil significantly higher than those of Cobrançosa and granitic soil, respectively. The highest soil K values were found in the LMg treatment and the lowest in the Con- treatment where K was not applied. Exchangeable Ca and Mg were significantly higher in the soil where Cobrançosa was grown and also in the schist soil. Exchangeable Ca levels in the soil were particularly high in the pots receiving CaCO_3_. The levels of exchangeable Mg in the soil were, in turn, the lowest in those pots. The comparison of CEC between cultivars and soil type followed the pattern of exchangeable Ca and Mg; between treatments, they followed mainly the pattern of exchangeable Ca, the most abundant base in the exchangeable complex. Extractable B was significantly higher in the pots grown with Cobrançosa and in the granitic soil. Regarding soil treatment, soil B levels were particularly high in the LMg treatment, where B uptake was apparently blocked. Soil Mn levels were significantly higher in Cobrançosa compared to the Arbequina pots and in schist in comparison to granite. Fertilizer treatments receiving CaCO_3_ (LMg and Con+) showed higher average values of Mn in the soil. Extractable Al was significantly lower in the treatments receiving CaCO_3_ (LMg and Con+).

### 2.4. Leaf Gas Exchange and Leaf Mass per Area

The Cobrançosa leaves presented significantly higher A, g_s_ and C_i_/C_a_ and lower A/g_s_ values than Arbequina (Table 5). Regarding the effect of soil type, plants that grew in schist-derived soil showed superior A, g_s_ and A/g_s_ and inferior C_i/_C_a_ values than plants that grew in granite soil. Leaf gas exchange traits also varied significantly between fertilizer treatments. The treatments that received B (+B and Con+) presented higher A/g_s_ and lower C_i_/C_a_ ratio, whereas stomatal conductance was larger in Con- and +B treatments and the uppermost net photosynthesis was observed in +B trees. On the other hand, the LMg treatment stands out negatively, as showed the lowest g_s_, A and A/g_s_ and the highest C_i_/C_a_ values. In general, except for the g_s_ value in Con- plants reported above, +P and Con- treatments presented intermediate A, g_s,_ A/g_s_ and C_i_/C_a_ values between those of the most productive (+B and Con+) and the least productive (LMg) treatment.

The morphological and structural leaf traits (individual leaf area and LMA) registered significant differences between cultivars, soils and fertilizer treatments (Table 6). The ILA and LMA traits were higher in the Cobrançosa cultivar compared to Arbequina, whereas those values were superior in schist and granite soil, respectively. Regarding the fertilizer treatments, the ILA tended to be higher in the most productive treatments and the LMA values varied in the opposite direction, showing a tendency to be higher in the less productive treatments.

## 3. Discussion

### 3.1. Cultivars Showed Differences in Precocity, Nutrient Concentrations, LMA and Physiological Performance

Cobrançosa started bearing fruit earlier but did not yield more total biomass than Arbequina. Cobrançosa is a larger-sized cultivar than Arbequina, the latter being frequently grown in hedgerow systems, with precocity being an important trait of the cultivar given the high financial input involved [20,21]. However, Cobrançosa passed the juvenile period even more quickly than Arbequina, having started producing fruit in the second year of growth, which seems to be an important characteristic of this cultivar to highlight.

Cobrançosa tended to show lower concentrations of some macronutrients in the leaves (N, P and Mg) and higher concentrations in the roots than Arbequina. This may be a result of the remobilization of nutrients to the fruits, which occurs mainly from the leaves [22]. Moreover, the higher degree of sclerophylly of Cobrançosa leaves expressed by the superior LMA (+36.3%) may also be involved in these responses, as LMA values generally show an inverse correlation with the mineral’s concentrations [23,24,25]. As pointed out by Bussoti et al. [23], a lower nutrient concentration in sclerophyllous leaves may not indicate poor nutrient status, suggesting that minerals are simply more diluted as higher sclerophylly implies a greater metabolic activity in producing structural carbon compounds, generally to overcome stress conditions. Confirming this premise, previous studies demonstrated that Cobrançosa is a cultivar better adapted to drought stress [26]. On the other hand, Cobrançosa is very popular in marginally cultivated regions, well adapted to rainfed agriculture and acidic, P-poor soils, often presenting low levels of macronutrients in the leaves without this apparently affecting its productivity [13]. In the Cobrançosa cultivar, a tendency towards the accumulation of nutrients in the roots, when available in the soil, mainly P, was observed, with the roots appearing to function as a buffer for the concentration of nutrients in the leaves [27].

Regarding micronutrients, perhaps the most relevant was the observation in Cobrançosa of lower Fe and higher Mn levels than in Arbequina, found consistently in all tissues. Arbequina cultivation is common in arid and semi-arid regions of Southern Europe [20], where neutral to alkaline soils predominate, a condition that reduces the availability of Fe in the soil [4,5]. Perhaps this is why Arbequina is efficient at absorbing Fe, as is the case of many other calcicolous species and cultivars adapted to alkaline soils [28]. For its part, Cobrançosa is commonly grown in acidic soils, with high levels of available Mn. High levels of Mn were also observed in the leaves of other acidophilic species in the region [12] and, perhaps for this reason, nutrient concentrations in Cobrançosa were higher than in Arbequina.

In terms of functional leaf characteristics, expressed by leaf gas exchange variables, Cobrançosa showed higher net photosynthesis due to superior stomatal conductance, probably as a result of lower leaf area per plant, which provides more water per unit of leaf area. In fact, considering the values of leaf dry weight and LMA presented in Figure 1 and Table 6, respectively, we estimate that the mean leaf area per plant was 8.16 dm^2^ in Cobrançosa and 10.45 dm^2^ in Arbequina. Furthermore, our results suggest that the variations in leaf mineral concentrations between cultivars did not have a significant effect on the photosynthetic rate and that in the present growing conditions the integration of all leaf gas exchange data allows us to infer that Arbequina leaves did not present more biochemical limitations to the photosynthetic process than Cobrançosa.

### 3.2. Schist and Granite Soils Showed Different Nutrient Bioavailability and Photosynthetic Activity

Plants grown in schist soil recorded higher total DMY and individually in each plant parts, roots, stems, leaves and fruits, the latter being absent in Arbequina, being these responses associated with the significant larger leaf area per plant (+62.7%), estimated as 11.27 and 6.93 dm^2^ by the methodology described in Section 4.1, and net photosynthesis (+21.1%). Plants grown in schist showed higher N concentrations in all tissues than those grown in granite soil. The schist soil had a higher organic matter content than the granite soil at the beginning of the experiment. Soil aeration, increased temperature and water availability are the main factors that activate soil microbiology and stimulate the mineralization of organic matter [5,29]. In this experiment, soil sampling and sieving, greenhouse cultivation and regular irrigation will have contributed to enhancing the mineralization of organic matter, with the increase in N supply being the most likely cause for the significant effect of schist soil to the observed increase in DMY. At the same time, plants grown in schist soil had higher Mn levels in all tissues than plants grown in granite soil. Acidic soils can present large amounts of Mn in solution, especially for a pH level below 5 [4,28]. When pH increases, the ionic form changes first to the hydroxide ion and, finally, to the insoluble oxide of the element [5]. In this region, due to the usually very low pH of the soils, very high levels of Mn have been observed in the tissues of cultivated plants [12]. As the initial pH was slightly lower and Mn levels were higher in schist soil (Table 7), this seems to be the most obvious reason to justify the higher Mn values in the tissues of plants grown in schist soil. With the range of Mn values found in this study, especially in leaves, we believe that the higher Mn concentrations will also have contributed to the improvement of photosynthetic activity and productivity. Mn plays a role in diverse processes of a plant’s life cycle such as the water-splitting reaction in photosystem II, the first step of photosynthesis, as well in respiration, scavenging of reactive oxygen species and hormone signaling [30]. The superior concentration of Mg and Zn in the leaves of plants that grew in the schist soil also deserve to be highlighted in the performance of olive trees. Magnesium is required for chlorophyll formation, playing a key role in the structure and photochemical activities of photosystems, photosynthetic electron transport and ribulose-1,5-bisphosphate carboxylase/oxygenase activity, being also involved in carbohydrate transport from source-to-sink organs [31]. In turn, zinc plays fundamental roles in various physiological and molecular mechanisms such as plant water relations, cell membrane stability, osmolyte accumulation, stomatal regulation, water use efficiency, photosynthesis, hormonal balance and antioxidant system, thus resulting in significantly better plant performance [32].

Plants grown in schist soil had lower P levels in all tissues. At the beginning of the experiment (Table 7), both soils had low P content, a common characteristic of these soils in the region [12,14]. Perhaps the result is due to a dilution effect, resulting from an equivalent availability of P in both soils and a greater production of biomass in the schist soil. The nutrient dilution/concentration effect is a common phenomenon that occurs when any agro-environmental factor other than the availability of a nutrient in the soil causes some change in DMY [33]. It could also be hypothesized that granite soil has greater P bioavailability than schist soil. However, as far as we know, there is no previous experimental evidence comparing these soils that proves this second hypothesis.

Plants grown in schist soil had lower B concentrations in leaves and roots than those grown in granite soil. In this region, both schist and granite soils provide little B to plants [12,16,17]. As schist soils gave rise to greater DMY, perhaps there was some dilution effect, as mentioned for P. However, the schist soil had not been cultivated for some time and the granite soil was taken from a vineyard, which is one of the crops where farmers most regularly apply B as a fertilizer [29]. Thus, initial B contents were, unsurprisingly, higher in the granite soil (Table 7) and this contributed to the higher tissue B concentrations in plants grown in this soil.

Most of the soil properties determined at the end of the study showed the trend observed in the determinations of the initial soil samples, with a higher content of organic matter, extractable K and exchangeable Ca and Mg, for example, in schist soil and a higher content of B, for example, in granite soil. Thus, cultivation will not have influenced much on the properties of the soil. However, the evolution of P levels in the soil deserves some attention. The initial P content in the soils were low and not very different in the two soils (Table 7). At the end of the experiment, P levels appeared higher in the granite soil. Although the schist soil resulted in a higher DMY, which could have led to a greater P uptake, the observed difference suggests that there may be other causes. Most plants, especially trees and shrubs, are known to develop mutualistic associations with mycorrhizal fungi [27,34]. Although mycorrhizal fungi can be associated with numerous benefits for plants, the most abundantly documented is the increase in P bioavailability [27,28]. It is expected that aspects associated with plant mycorrhization have promoted P solubility in the less acidic granite soil and help to justify the differences observed in P levels in the two soils. Root colonization with mycorrhizae is an important aspect in the adaptation of plants to acidic mineral soils with low P availability and high Al content [28,35].

The joint analysis of all leaf gas exchange data revealed that plants that grew in the schist soil showed greater photosynthetic performance due to lower non-stomatal limitations, largely determined by better nutritional status, as previously mentioned, namely the higher N, Mg, Mn and Zn levels in leaves, but also due to inferior stomatal limitations, an indication of foremost water status. The last reason is interesting, given that the leaf area per plant was significantly higher in the plants that grew in the schist soil, as mentioned before. The higher organic matter, clay and silt levels and the lower sand content contributed to greater water holding capacity of the schist soil, while the greater biomass of the root system allows the enhancement of water absorption.

### 3.3. LMg Treatment Drastically Reduced Plant Growth by Preventing B Uptake

The +B and Con+ treatments gave rise to higher DMY than the other treatments and were the only treatments bearing fruit. These two treatments have in common the application of B, which made possible to increase the leaf area per plant to 12.59 and 12.93 dm^2^, respectively, while in the other treatments the values were 9.48, 6.89 and 3.75 dm^2^ in +P, Con- and LMg, respectively. In addition, the less productive treatments also presented lower net photosynthetic rate and, in general, higher LMA. The lack of nutrients will decrease growth more than photosynthesis, leading to accumulation of total nonstructural carbohydrates, which may increase LMA [24]. Taking into account that in the treatments that did not receive B, the average levels of the nutrient in the leaves were low (12.6 to 17.2 mg kg^–1^), when compared with the values of the sufficiency range for mature olive trees (20–75 mg kg^–1^ in the summer season) [36], it is likely that B availability had a major influence on the difference in DMY, plant leaf area and leaf gas exchange traits observed in the different treatments. B is a nutrient of particular importance for dicotyledonous, being required in greater amounts than any other micronutrient, playing an important role in the cell wall biosynthesis and in several metabolic pathways such as amino acid and protein metabolism [37,38]. B deficiency also results in a decrease in the reproductive success because of poor flower production and pollen viability. The reproductive failure can be observed even without deficiency symptoms in the foliage, suggesting that the B requirement for the reproductive process is greater than for vegetative tissues [38]. Therefore, it seems unequivocal that B played a determining role in the presence of fruits on plants. The results of biomass production and reproductive success of B treatments are in line with those of many other studies that highlighted the importance of B for the cultivation of dicots in the region [12,15,16,17].

The LMg treatment resulted in lower DMY, leaf area and net photosynthesis than Con-. This result is unexpected, considering that increasing pH and supplying Ca and Mg to acidic soils are the main aspects of liming that normally justify an increase in DMY [6,10]. In the LMg treatment, all growth tips died, and chlorosis appeared in the apical part of the basal leaves, typical symptoms of severe B deficiency [39]. In this treatment, the biomass in the root system was a particularly low response that compromises the absorption of water and minerals. In terms of leaf gas exchange traits, LMg trees registered the lowest values of A, g_s_ and A/g_s_ and the highest of Ci/Ca. Which means that, apart from the stomatal limitations, several non-stomatal components negatively influenced net photosynthetic productivity and consequently all plant growth and development [40]. The negative influence of B deficiency on A and g_s_ was reported from previous studies [41,42,43,44]. B availability is related to soil pH. The element is more available in acidic soils, with deficiency being common at high pH levels in sandy soils due to their low B content [5,38]. At a high pH, B is quite tightly bound, especially between pH 7 and 9, which is the range of lowest availability of the element [5,18]. In addition, plants tend to have a greater need for B if Ca is abundant [5]. Furthermore, at alkaline pH levels, B is adsorbed by organic colloids with a binding strength even greater than that of inorganic colloids [5]. In the LMg treatment the pH reached 7.36, which constitutes a situation of overliming, and was the cause of the severe B deficiency. However, in the Con+ treatment the pH reached an even higher value (7.48), and this treatment was associated with high DMY. The B applied in this treatment appears to have been sufficient to counterbalance the strong reduction in nutrient availability caused by the increase in pH and Ca supply.

The Con- treatment resulted in lower DMY than the other treatments, excluding LMg. Con- was the only treatment that did not receive N. When the effect of the soils was compared, it became clear that it was the difference in N availability that led to greater DMY in the schist soil. On the other hand, it can be observed that in the Con- treatment the N levels in the tissues were very low, and in the case of leaves, well below the sufficiency range established for mature trees [36]. Therefore, it seems clear that it was the lower availability of N in the soil that led to the low DMY in this treatment. N is an ecological factor determining agricultural productivity, and it is also common to observe a response to the application of the nutrient in field and pot experiments carried out on olive [45,46]. It should be noted that Con- treatment did not present stomatal limitations to photosynthesis, a fact that is associated with the low size of the leaf area and the large accumulation of biomass in root system, the second in absolute terms, right after the +B treatment. However, Con- treatment showed lower intrinsic water use efficiency than the more productive treatments, aspect associated with non-stomatal limitations for photosynthesis, probably related with the low N and Zn concentrations in the leaves.

The +P treatment resulted in plants with a lower concentration of B in the leaves than the LMg treatment itself, without such a sharp drop in DMY. This phenomenon has been called the Piper-Steenbjerg effect in the literature and represents situations in which the supply of a nutrient, previously in a situation of severe deficiency, stimulates plant growth at a rate higher than the uptake of the nutrient, leading to its dilution in the tissues [33,47]. As far as we know, for B the effect has only been described for sugar beet [48]. The result observed in this experiment may have the following explanation. In the LMg treatment, all new shoots dried due to a lack of B, leaving old leaves, some already present on the cuttings, with B concentrations that were not so low, as a result of reduced B mobility in the plant. B is a unique nutrient with regard to its mobility in plants. It appears to have restricted mobility in several species due to restricted mobility in the phloem, while being quite mobile in others [38,49]. In olive trees, a study with the Manzanillo cultivar showed some B mobility in plant tissues [50], while in other studies it appears that B mobility depends on the cultivar [15]. This study seems to show that for the cultivars Cobrançosa and Arbequina the mobility of B is quite low.

## 4. Materials and Methods

### 4.1. Experimental Design and Characterization of Factors and Treatments

The experiment was arranged into a factorial with three factors or independent variables (three-way ANOVA): (i) soil type, two levels [schist (Sch) and granite (Gra)]; (ii) cultivars, two levels [Cobrançosa (Cob) and Arbequina (Arb)]; and (iii) fertilizer treatments, five levels [liming (CaCO_3_) plus Mg (LMg), P application (+P), B application (+B), all the fertilizing materials (Con+), and an untreated control (Con-)]. Thus, the five fertilizer treatments were applied to each of the soils, which, in turn, were grown with the two varieties, in a unique experiment conducted at the same time.

Schist soil was collected in Mirandela (41°26′40.4″ N 7°14′20.9″ W), NE Portugal. This soil was selected because it is one of the predominant soil types in the region and has a very low pH. The plot was fallow for eight years, after a long period of cultivation in a biannual wheat-fallow rotation. On the sampling date, the farmer was preparing the plot for planting an almond orchard. The soil used in the pot experiment was collected from several random points on the plot, at a depth of 0–20 cm. After harvesting, the soil was adequately homogenized, sieved (2 mm) and oven dried (40 °C). The soil is classified as a Dystric Leptosol [51], has a loamy-sand texture, and a pH of 4.53. Some of the soil properties determined at the beginning of the experiment are presented in Table 7. The granite soil was collected in Valpaços (41°36′32.2″ N 7°20′09.2″ W), also in NE Portugal. It was chosen for this experiment because it belongs to one of the main lithological formations in the region, granite, and had a very acidic pH (4.86). On the sampling date, the plot was cultivated with vines and the soil was managed with conventional tillage. It is also a Dystric Leptosol soil [51] with a sandy texture. Other soil properties at the sampling time are presented in Table 7.

The Cobrançosa cultivar is the most popular of those grown in NE Portugal and is recognized as a cultivar which is well-adapted to these acidic soils. It is used mainly in traditional rainfed managed olive groves typically spaced 7 m × 7 m. The other cultivar, Arbequina, is very popular in southern Spain, being a cultivar typically well adapted to neutral to alkaline soils. It is a cultivar that has been widely used in olive groves in high-density hedgerows and in irrigation. In the experiment, pre-rooted cuttings were used, pruned to a simple stem with ~20 cm in height.

The main objective of the treatment was to raise soil pH, simulating the agricultural practice of liming. To raise the pH to an estimated value close to 7, laboratory CaCO_3_ was used, which is of greater purity than agricultural limestone. Considering the buffer capacity of the soil, which is very dependent on its texture and organic matter content, it was estimated that 4 and 5 t ha^−1^ of CaCO_3_ would be needed in granite and schist soils, respectively. To estimate the amount of CaCO_3_ to be used in the pots, it was considered that the arable layer (20 cm) in 1 ha has a mass of ~2000 t of soil (<2 mm) [29]. Thus, 6 and 7.5 g pot^−1^ of CaCO_3_ were used in granite and schist soils, respectively, taking into account that each pot (~3 L) was filled with 3 kg of dry and sieved soil (2 mm mesh). The amendment was added to the pots, properly mixed with the soil just before planting.

To provide Mg, P and B to treatments that received these nutrients, a different approach was adopted. We sought to provide a certain amount of nutrient to each of the plants, based on the rates that are normally used per ha. Thus, as the plants grown in the pots are somewhat similar in size to an annual herbaceous plant, to estimate the amount of nutrient to be applied, a density of 120,000 plants ha^−1^ was assumed (corresponding in part to the distance between the pots). Thus, rates equivalent to 15, 25 and 1.5 kg ha^−1^ (125.0, 208.3 and 12.5 mg pot^−1^) of Mg, P and B were added, respectively. The fertilizers magnesium chloride (MgCl_2_.6H_2_O), orthophosphoric acid (H_3_PO_4_) and boric acid (H_3_BO_3_) were used. To apply Mg, 209.1 g L^−1^ of MgCl_2_ were used, each ml containing 25 mg of Mg. Each pot received 5 mL of solution. To apply P, a solution with 131.5 g L^−1^ of H_3_PO_4_ was used. The pH of this solution (1.6) was adjusted to 6 using 300 mL of 1 M sodium hydroxide (NaOH), leaving the final volume at 1300 mL. Each ml of solution contained 32 mg of P. Each pot received 6.5 mL of solution. For B, 14.3 g L^−1^ of H_3_BO_3_ was used, each ml containing 2.5 mg of B. Each pot received 5 mL of solution. All these solutions were applied shortly after planting the cuttings, with the application repeated one year later.

All treatments, except for Con-, received N and potassium (K). As N and K are important plant growth factors, but generally less related to soil acidity, it was decided to apply them in all fertilized treatments so that the lack of these nutrients did not prevent the plants’ response to the fertilizer treatments of the experimental design. When applying N and K, the principles established for Mg, P and B were followed. N and K were applied at doses of 50 kg ha^−1^ (416 mg ha^−1^ of N) and 30 kg ha^−1^ (250 mg pot^−1^ of K), respectively. N was applied in the form of ammonium nitrate (NH_4_NO_3_), at a concentration of 83.3 g L^−1^, each ml containing 83.2 mg of N. Each pot received 5 mL of solution. Unlike other nutrients, N was applied twice a year, due to the high risk of loss through denitrification. To prepare the K solution, potassium chloride (KCl) was used in a proportion of 94.87 g L^−1^ (each ml with 50 mg of K) and 5 mL was applied to each pot. The solution containing K was applied only once a year.

### 4.2. Pot Management

The cuttings were planted on 16 November 2020. After planting, the pots were placed in a double-walled polycarbonate greenhouse, with side and overhead openings to ensure adequate ventilation. Even though the conditions inside the greenhouse were relatively homogeneous, with diffuse radiation due to the cover, the individual position of the pots in the experimental layout was changed biweekly.

After planting the cuttings, the emergence of weeds was also monitored, which were immediately pulled out to prevent nutrient uptake. Throughout the experimental period, the plants were watered regularly whenever necessary. The frequency of irrigation and the volume of water used varied over time, regarding environmental conditions, namely temperature, but also with regard to the size of the plants, since these are the variables that cause evapotranspiration to vary. Initially, 150 mL of water was applied to each irrigation in all pots. Solutions with the nutrients referred to in the experimental design were applied to the pots immediately before watering to facilitate the movement of nutrients into the root zone.

### 4.3. Leaf Gas Exchange and Leaf Mass per Area

Leaf gas exchange data were acquired one month before the end of the experiment, during the morning period (10:00–12:00 local time), using two portable LCPro+ infrared gas analyzers (ADC Bioscientific Ltd., Hoddesdon, UK) coupled to a broad leaf chamber, and with a LED light unit that provided photosynthetic photon flux density of 1000 µmol m^−2^ s^−1^. Five plants were used per condition, and in each plant, measurements were performed in two fully developed and well-exposed healthy leaves, making up ten biological replicates per experimental condition. The net CO_2_ assimilation rate (A), intercellular to atmospheric CO_2_ concentration ratio (C_i_/C_a_), and stomatal conductance (g_s_) were estimated according to von Caemmerer and Farquhar [52]. The intrinsic water use efficiency was calculated as the A/g_s_ ratio. After the leaf gas exchange measurements, the same leaves were collected and the following traits were assessed: individual leaf area (ILA), measured with the WinDIAS leaf image analysis system (Delta-T Devices, Cambridge, UK), and dry weight (DW), after drying in a force-draft oven at 60 °C to a constant weight. Then, leaf mass per area (LMA, g m^−2^) was calculated as DW/ILA ratio.

### 4.4. Cutting of Plants and Sample Preparation

The experiment ended on 13 October 2022, almost two years after it began. The plants were removed from the pots with the soil adhering to the root system. The soil was carefully separated from the roots, to avoid breaking them, and homogenized appropriately, after which a sample weighing ~250 g was sent to the laboratory. The plants were separated into roots, stems, leaves and fruits. The roots were washed before being sent to the laboratory. A sieve (1 mm mesh) was used to avoid loss of roots, and washing was carried out in running water at low pressure.

The soil samples were oven dried at 40 °C and sieved again through a 2 mm mesh. The plant tissue samples (roots, stems, leaves and fruits) were also oven dried at 70 °C until constant mass and weighed to obtain the DMY in each part of the plant. Roots, leaves and stems were ground in a mill using a 1 mm mesh. The fruits were not analyzed because only part of the plants bore fruits.

### 4.5. Soil and Tissue Analyses

Initial soil samples and those collected at the end of the pot experiment were analyzed for pH (H_2_O and KCl) (soil:solution, 1:2.5), easily oxidizable carbon (C) (Walkley–Black), extractable P and K (Egner–Riehm method), cation-exchange capacity (ammonium acetate, pH 7.0) and extractable B (hot-water and azomethine-H). Soil separates were determined by the Robinson pipette method but only from the initial soil samples. For more details on these analytical procedures, the reader is referred to Van Reeuwijk [53]. The availability of other elements (Cu, Fe, Zn, Mn and Al) in the soil was determined by atomic absorption spectrometry after extraction with DTPA (diethylenetriaminepentaacetic acid) buffered at pH 7.3, following the standard procedure of FAO [54].

Tissue samples (roots, stems and leaves) were used to determine the elemental composition. N was determined by the Kjeldahl method, B and P by colorimetry and K, Ca, Mg, Mn, Fe, Cu, Zn and Al by atomic absorption spectrophotometry [55] after the samples have been digested with nitric acid in a microwave oven. Al was not determined in the stems and leaves, since plants tend to have exclusion mechanisms for this element accumulating it only in the roots [28].

### 4.6. Data Analysis

All variables considered relevant for this study were tested for normality and homogeneity of variances using the Shapiro–Wilk test and Bartlett’s test, respectively. Thereafter, a comparison of the effect of the treatments was provided by three-way ANOVA. When significant differences between treatments were found (*p* < 0.05), the means were separated by the multiple range Tukey HSD test (*α* = 0.05).

## 5. Conclusions

The Cobrançosa cultivar started bearing fruit sooner than Arbequina, a beneficial characteristic in any cultivar, as it allows an earlier economic return from any planting project.

DMY, leaf area and net photosynthesis were higher in the plants grown in schist soil than in those grown in granite soil. Schist soil had a higher organic matter content, and the experimental conditions favored the mineralization of organic matter, which would have released more N for the plants. The concentration of N and other nutrients in the tissues made it clear that the increase in N availability in the soil was the most important contribution of organic matter mineralization to plant growth and development.

The LMg treatment drastically reduced DMY, leaf area and A, registering significantly lower values than the unfertilized control. Raising the pH level above 7 (7.36) will have reduced the bioavailability of B in these sandy soils already poor in the nutrient, which induced a severe B deficiency. In the Con+ treatment, which also raised the soil pH above 7 (7.48), there was no yield loss. The B provided in this treatment prevented nutrient deficiency, appearing as one of the most productive treatments together with the +B treatment. It became clear that in these soils the management of B availability is decisive for olive performance, a dicotyledonous species. Furthermore, in sandy, B-poor soils, the application of limestone should not excessively increase the pH, and the use of lime should be complemented with a reinforced application of B.

## Figures and Tables

**Table 1:** Nutrient concentration in the leaves of olive plants of the cultivars Cobrançosa (Cob) and Arbequina (Arb), grown in schist (Sch) and granite (Gra) derived soils under five fertilizer treatments [untreated control (Con-), boron (+B) and phosphorus (+P) application, calcium carbonate plus magnesium application (LMg), and a positive control receiving all these fertilizers (Con+)]. Within cultivar, soil or treatment, means followed by the same letter are not significantly different by the Tukey HSD test (α = 0.05).

		Nitrogen	Phosphorus	Potassium	Calcium	Magnesium		Boron	Iron	Manganese	Zinc	Copper
		(g kg^−1^)						(mg kg^−1^)				
Cultivar (C)	Cob	15.80 b	1.44 b	8.90 a	5.55 a	0.89 b		18.5 a	206.1 b	70.7 a	15.3 a	4.5 b
	Arb	17.03 a	1.80 a	9.33 a	4.99 a	1.05 a		19.0 a	549.5 a	54.1 b	16.5 a	5.5 a
Soil (S)	Sch	18.01 a	1.46 b	9.09 a	5.12 a	1.11 a		17.4 b	378.2 a	80.3 a	18.9 a	4.9 a
	Gra	14.83 b	1.78 a	9.14 a	5.42 a	0.82 b		20.2 a	377.4 a	44.5 b	12.9 b	5.1 a
Treatment (T)	Con-	11.74 c	1.72 a	9.28 a	4.42 c	0.88 a		17.2 b	419.1 a	62.2 b	12.7 c	5.5 b
	+B	16.15 b	1.43 a	9.68 a	4.37 c	1.03 a		25.0 a	473.3 a	81.9 a	19.1 a	4.6 c
	+P	18.48 a	1.76 a	9.62 a	4.10 c	0.95 a		12.6 c	420.9 a	80.7 a	17.2 a	7.3 a
	LMg	18.87 a	1.52 a	8.56 a	7.56 a	1.06 a		15.0 c	315.6 b	42.0 c	13.7 bc	4.4 c
	Con+	16.85 ab	1.68 a	8.43 a	5.90 b	0.93 a		23.6 a	260.0 b	45.1 c	16.9 ab	3.1 d
	P (C)	0.0112	<0.0001	0.2004	0.2677	0.0005		0.9568	<0.0001	<0.0001	0.1025	<0.0001
	P (S)	<0.0001	0.0006	0.8714	0.3846	<0.0001		<0.0001	0.9518	<0.0001	<0.0001	0.2676
	P (T)	<0.0001	0.2150	0.0545	<0.0001	0.0535		<0.0001	<0.0001	<0.0001	<0.0001	<0.0001
	P (C × S)	0.1042	0.0466	0.8617	0.4501	0.5327		0.0024	<0.0001	<0.0001	<0.0001	0.0180
	P (C × T)	<0.0001	0.2150	0.0189	0.0137	0.2308		<0.0001	<0.0001	<0.0001	<0.0001	0.0012
	P (S × T)	0.0013	0.9114	0.0033	0.3159	0.3103		<0.0001	<0.0001	<0.0001	<0.0001	<0.0001
	P (C × S x T)	0.0178	0.7980	0.0104	0.3113	0.3356		<0.0001	<0.0001	0.0004	<0.0001	0.7936

**Table 2:** Nutrient concentration in the stems of olive plants of the cultivars Cobrançosa (Cob) and Arbequina (Arb), grown in schist (Sch) and granite (Gra) derived soils under five fertilizer treatments [untreated control (Con-), boron (+B) and phosphorus (+P) application, calcium carbonate plus magnesium application (LMg), and a positive control receiving all these fertilizers (Con+)]. Within cultivar, soil or treatment, means followed by the same letter are not significantly different by the Tukey HSD test (α = 0.05).

		Nitrogen	Phosphorus	Potassium	Calcium	Magnesium		Boron	Iron	Manganese	Zinc	Copper
		(g kg^−1^)						(mg kg^−1^)				
Cultivar (C)	Cob	8.71 a	1.26 b	7.00 a	5.30 a	0.63 b		15.8 b	284.3 b	40.6 a	16.3 b	3.7 b
	Arb	8.23 b	1.36 a	6.59 a	4.74 b	0.72 a		19.2 a	948.8 a	41.7 a	26.1 a	8.7 a
Soil (S)	Sch	9.03 a	1.26 b	6.96 a	5.56 a	0.73 a		16.5 b	576.5 b	48.0 a	19.7 b	4.8 b
	Gra	7.91 b	1.36 a	6.64 a	4.48 b	0.62 b		18.5 a	656.6 a	34.2 b	22.7 a	7.7 a
Treatment (T)	Con-	6.62 d	1.27 a	6.55 a	4.37 b	0.66 a		15.9 a	927.7 a	48.7 a	24.2 a	6.4 ab
	+B	8.14 c	1.27 a	7.11 a	4.66 b	0.67 a		21.1 a	459.8 b	55.2 a	27.0 a	6.1 ab
	+P	9.79 a	1.41 a	7.19 a	4.89 b	0.74 a		13.7 c	976.6 a	50.8 a	23.3 a	6.9 a
	LMg	9.07 ab	1.31 a	6.48 a	5.88 a	0.69 a		17.3 b	300.8 c	20.2 c	13.2 c	6.3 ab
	Con+	8.73 bc	1.29 a	6.65 a	4.94 b	0.61 a		19.6 a	417.6 bc	30.7 b	18.2 b	5.4 b
	P (C)	0.0050	0.0030	0.0470	0.0008	0.0018		<0.0001	<0.0001	0.5992	<0.0001	<0.0001
	P (S)	<0.0001	0.0042	0.2197	<0.0001	0.0006		<0.0001	0.0197	<0.0001	0.004	<0.0001
	P (T)	<0.0001	0.0599	0.2004	<0.0001	0.0733		<0.0001	<0.0001	<0.0001	<0.0001	0.0250
	P (C × S)	0.0088	0.0008	0.8658	0.0031	0.3129		0.3987	<0.0001	0.0117	0.6237	<0.0001
	P (C × T)	<0.0001	0.1135	0.1213	0.0026	0.1658		<0.0001	<0.0001	0.0005	0.0063	<0.0001
	P (S × T)	<0.0001	0.3016	0.6823	0.0872	0.3159		0.0023	<0.0001	0.0139	0.064	<0.0001
	P (C × S × T)	0.5308	0.0388	0.0159	0.0011	0.0688		<0.0001	<0.0001	0.0014	<0.0001	<0.0001

**Table 3:** Nutrient concentration in the roots of olive plants of the cultivars Cobrançosa (Cob) and Arbequina (Arb), grown in schist (Sch) and granite (Gra) derived soils under five fertilizer treatments [untreated control (Con-), boron (+B) and phosphorus (+P) application, calcium carbonate plus magnesium application (LMg), and a positive control receiving all these fertilizers (Con+)]. Within cultivar, soil or treatment, means followed by the same letter are not significantly different by the Tukey HSD test (α = 0.05).

		Nitrogen	Phosphorus	Potassium	Calcium	Magnesium		Boron	Iron	Manganese	Zinc	Copper	Aluminum
		(g kg^−1^)						(mg kg^−1^)					
Cultivar (C)	Cob	15.66 a	1.66 a	6.81 a	5.57 a	2.19 a		17.4 a	2117.1 b	236.8 a	28.5 a	25.5 b	2188.4 a
	Arb	13.09 b	1.33 b	6.38 a	4.94 b	1.67 b		16.7 a	3071.7 a	203.5 b	23.8 b	29.1 a	2118.6 a
Soil (S)	Sch	15.25 a	1.28 b	6.68 a	5.34 a	1.95 a		16.8 a	2505.1 a	257.2 a	18.0 b	19.0 b	2123.8 a
	Gra	13.50 b	1.71 a	6.51 a	5.18 a	1.92 a		17.3 a	2683.7 a	183.1 b	34.3 a	35.6 a	2183.2 a
Treatment (T)	Con-	10.81 c	1.56 a	7.05 ab	4.47 c	1.99 ab		13.5 d	3029.9 a	279.2 a	41.6 a	45.3 a	2428.1 a
	+B	13.37 b	1.41 a	5.74 b	4.21 c	1.76 b		18.6 b	2299.5 c	287.8 a	26.6 c	22.5 bc	2325.8 a
	+P	15.99 a	1.50 a	6.59 ab	4.46 c	1.74 b		13.1 d	2876.3 ab	243.8 a	29.8 b	24.9 b	2231.7 a
	LMg	17.22 a	1.60 a	7.20 a	7.07 a	1.97 ab		16.6 c	2227.7 c	111.0 c	14.5 e	20.0 c	1839.1 b
	Con+	14.49 b	1.40 a	6.39 ab	6.07 b	2.21 a		23.5 a	2538.8 bc	179.1 b	18.4 d	23.9 b	1942.8 b
	P (C)	<0.0001	<0.0001	0.1828	0.0014	<0.0001		0.0663	<0.0001	0.003	<0.0001	<0.0001	0.3559
	P (S)	<0.0001	<0.0001	0.596	0.3855	0.7194		0.6533	0.0700	<0.0001	<0.0001	<0.0001	0.4309
	P (T)	<0.0001	0.1642	0.0465	<0.0001	0.0166		<0.0001	<0.0001	<0.0001	<0.0001	<0.0001	<0.0001
	P (C × S)	0.0668	0.0024	0.4456	0.2155	0.5750		0.1592	<0.0001	<0.0001	<0.0001	0.8790	<0.0001
	P (C × T)	0.0021	0.6632	0.0415	0.0002	0.0211		0.0074	<0.0001	<0.0001	<0.0001	<0.0001	<0.0001
	P (S × T)	0.0095	0.0043	<0.0001	0.3494	0.5332		<0.0001	<0.0001	<0.0001	<0.0001	<0.0001	<0.0001
	P (C × S × T)	0.0014	0.3318	0.0026	<0.0001	0.1792		<0.0001	0.0003	0.0024	<0.0001	0.1904	<0.0001

**Table 4:** Selected soil properties recorded in the pots of the cultivars Cobrançosa (Cob) and Arbequina (Arb), grown in schist (Sch) and granite (Gra) derived soils under five fertilizer treatments [untreated control (Con-), boron (+B) and phosphorus (+P) application, calcium carbonate plus magnesium application (LMg), and a positive control receiving all these fertilizers (Con+)]. Within cultivar, soil or treatment, means followed by the same letter are not significantly different by the Tukey HSD test (α = 0.05).

		Organic C		Extract P	Extract K	Exch Ca^2+^	Exch Mg^2+^	CEC	Extract B	Extract Mn	Extract Al
		(g kg^−1^)	pH _(H2O)_	(mg kg^−1^, P_2_O_5_)	(mg kg^−1^, K_2_O)	(cmol_+_ kg^−1^)			(mg kg^−1^)		
Cultivar (C)	Cob	5.3 a	6.5 a	78.0 a	84.6 b	5.4 a	2.2 a	8.0 a	0.8 a	20.6 a	35.9 a
	Arb	5.2 b	6.5 a	71.6 b	90.8 a	4.5 b	1.9 b	7.2 b	0.7 b	17.4 b	36.8 a
Soil (S)	Sch	8.6 a	6.5 a	59.2 b	103.7 a	5.3 a	2.2 a	8.3 a	0.6 b	32.9 a	37.4 a
	Gra	2.0 b	6.5 a	91.0 a	71.6 b	4.6 b	1.9 b	6.9 b	0.9 a	5.2 b	35.2 a
Treatment (T)	Con-	5.3 a	5.9 c	48.5 c	67.2 d	4.1 b	2.3 a	7.1 bc	0.6 c	17.4 b	39.2 a
	+B	5.1 a	5.8 d	47.2 c	79.4 c	3.6 c	2.2 a	6.6 c	0.5 c	14.7 b	39.9 a
	+P	5.2 a	5.8 cd	85.9 b	84.0 c	3.6 c	2.2 a	6.5 c	0.5 c	18.2 ab	40.3 a
	LMg	5.3 a	7.4 b	99.2 a	115.1 a	6.8 a	1.7 c	8.4 ab	1.1 a	22.6 a	30.2 b
	Con+	5.2 a	7.5 a	94.8 a	92.8 b	6.8 a	2.0 b	9.6 a	0.9 b	22.1 a	32.0 b
	P (C)	0.0047	0.0721	0.0002	<0.0001	<0.0001	<0.0001	0.0123	0.0075	0.0034	0.5825
	P (S)	<0.0001	0.3128	<0.0001	<0.0001	<0.0001	<0.0001	<0.0001	<0.0001	<0.0001	0.1515
	P (T)	0.1140	<0.0001	<0.0001	<0.0001	<0.0001	<0.0001	<0.0001	<0.0001	<0.0001	<0.0001
	P (C × S)	0.0084	0.1538	0.1579	0.5156	0.0008	<0.0001	0.0118	<0.0001	0.0036	0.0027
	P (C × T)	0.1521	0.9182	0.2587	<0.0001	<0.0001	<0.0001	0.4200	0.0060	0.1077	0.0007
	P (S × T)	0.1500	0.0881	0.0009	<0.0001	<0.0001	<0.0001	0.0222	<0.0001	0.0134	<0.0001
	P (C × S × T)	0.1066	0.1262	<0.0001	0.0007	<0.0001	<0.0001	0.0094	<0.0001	0.1020	0.0008

**Table 5:** Stomatal conductance (g_s_), net photosynthetic rate (A), intrinsic water use efficiency (A/g_s_) and intercellular to atmospheric CO_2_ concentration ratio (Ci/Ca) in olive leaves of the cultivars Cobrançosa (Cob) and Arbequina (Arb), grown in schist (Sch) and granite (Gra) derived soils under five fertilizer treatments [untreated control (Con-), boron (+B) and phosphorus (+P) application, calcium carbonate plus magnesium application (LMg), and a positive control receiving all these fertilizers (Con+)]. Within cultivar, soil or treatment, means followed by the same letter are not significantly different by the Tukey HSD test (α = 0.05).

		g_s_	A	A/g_s_	Ci/Ca
		mmol m^−2^ s^−1^	μmol m^−2^ s^−1^	µmol mol^−1^	
Cultivar (C)	Cob	135.92 a	10.09 a	72.87 b	0.67 a
	Arb	96.47 b	8.92 b	94.14 a	0.58 b
Soil (S)	Sch	125.78 a	10.40 a	85.64 a	0.62 b
	Gra	105.96 b	8.59 b	81.72 b	0.64 a
Treatment (T)	Con-	136.27 a	10.53 b	79.37 c	0.64 b
	+B	135.76 a	12.14 a	92.11 b	0.59 c
	+P	117.24 b	8.91 c	80.69 c	0.64 b
	LMg	79.69 c	5.24 d	65.75 d	0.71 a
	Con+	110.39 b	10.65 b	100.50 a	0.56 c
	P (C)	<0.0001	<0.0001	<0.0001	<0.0001
	P (S)	<0.0001	<0.0001	0.0210	0.0207
	P (T)	<0.0001	<0.0001	<0.0001	<0.0001
	P (C × S)	0.0288	0.2484	0.9912	0.9467
	P (C × T)	0.0002	0.0043	0.0343	0.0341
	P (S × T)	<0.0001	<0.0001	<0.0001	<0.0001
	P (C × S × T)	0.0015	0.0059	0.0005	0.0008

**Table 6:** Individual leaf area (ILA) and leaf mass per area (LMA) from olive plants of the cultivars Cobrançosa (Cob) and Arbequina (Arb), grown in schist (Sch) and granite (Gra) derived soils under five fertilizer treatments [untreated control (Con-), boron (+B) and phosphorus (+P) application, calcium carbonate plus magnesium application (LMg), and positive control receiving all these fertilizers (Con+)]. Within cultivar, soil or treatment, means followed by the same letter are not significantly different by the Tukey HSD test (α = 0.05).

		ILA	LMA
		cm^2^	g m^−2^
Cultivar (C)	Cob	3.99 a	192.63 a
	Arb	3.12 b	141.31 b
Soil (S)	Sch	3.80 a	165.28 b
	Gra	3.30 b	168.66 a
Treatment (T)	Con-	3.32 b	171.54 ab
	+B	3.69 ab	165.89 b
	+P	3.59 ab	158.27 c
	LMg	3.27 b	173.63 a
	Con+	3.89 a	165.52 b
	P (C)	<0.0001	<0.0001
	P (S)	<0.0001	0.0162
	P (T)	0.0050	<0.0001
	P (C × S)	0.2019	0.0195
	P (C × T)	0.3615	<0.0001
	P (S × T)	0.1609	0.0019
	P (C × S × T)	0.5400	<0.0001

**Table 7:** Selected soil properties (average ± standard deviation, n = 3) from composite samples (10 cores taken per composite sample) taken at 0–0.20 m depth at the beginning of the study.

Soil Properties	Schist	Granite
^1^ Organic carbon (g kg^−1^)	7.3 ± 0.93	1.8 ± 0.10
^2^ pH (H_2_O)	4.5 ± 0.13	4.9 ± 0.09
^3^ Extract. phosphorus (mg kg^−1^ P_2_O_5_)	27.6 ± 6.40	21.5 ± 5.12
^3^ Extract. potassium (mg kg^−1^, K_2_O)	148.8 ± 11.63	63.6 ± 5.13
^4^ Extract. boron (mg kg^−1^)	1.0 ± 0.16	1.7 ± 0.13
^5^ Extract. iron (mg kg^−1^)	34.0 ± 5.26	20.3 ± 2.99
^5^ Extract. manganese (mg kg^−1^)	39.6 ± 6.62	11.3 ± 1.28
^5^ Extract. zinc (mg kg^−1^)	2.0 ± 0.20	4.1 ± 0.27
^5^ Extract. copper (mg kg^−1^)	0.2 ± 0.03	0.6 ± 0.08
^6^ Exchang. calcium (cmol_c_ kg^−1^)	2.7 ± 0.27	1.9 ± 0.13
^6^ Exchang. magnesium (cmol_c_ kg^−1^)	0.9 ± 0.07	0.6 ± 0.10
^6^ Exchang. potassium (cmol_c_ kg^−1^)	0.3 ± 0.05	0.2 ± 0.03
^6^ Exchang. sodium (cmol_c_ kg^−1^)	0.3 ± 0.08	0.2 ± 0.03
^7^ Exchang. acidity (cmol_c_ kg^−1^)	2.3 ± 0.10	0.9 ± 0.10
^7^ Exchang. aluminum (cmol_c_ kg^−1^)	1.1 ± 0.15	0.8 ± 0.10
Cation exchange capacity (cmol_c_ kg^−1^)	6.4 ± 0.14	3.8 ± 0.12
^8^ Clay (g kg^−1^)	46.1 ± 6.55	24.8 ± 3.04
^8^ Silt (g kg^−1^)	156.7 ± 19.23	90.9 ± 10.31
^8^ Sand (g kg^−1^)	797.1 ± 25.30	884.3 ± 8.52

^1^ Wet digestion (Walkley-Black); ^2^ Potentiometry; ^3^ Ammonium lactate; ^4^ Hot water, azomethine-H; ^5^ ammonium acetate and EDTA; ^6^ Ammonium acetate; ^7^ Potassium chloride; ^8^ Robinson pipette.

## Data Availability

Data is contained within the article.
